# Improving Speaker Recognition by Biometric Voice Deconstruction

**DOI:** 10.3389/fbioe.2015.00126

**Published:** 2015-09-22

**Authors:** Luis Miguel Mazaira-Fernandez, Agustín Álvarez-Marquina, Pedro Gómez-Vilda

**Affiliations:** ^1^Neuromorphic Voice Processing Laboratory, Center for Biomedical Technology, Universidad Politécnica de Madrid, Madrid, Spain

**Keywords:** voice biometry, voice processing, speaker recognition, speaker characterization, source-tract separation, GMM–UBM

## Abstract

Person identification, especially in critical environments, has always been a subject of great interest. However, it has gained a new dimension in a world threatened by a new kind of terrorism that uses social networks (e.g., *YouTube*) to broadcast its message. In this new scenario, classical identification methods (such as fingerprints or face recognition) have been forcedly replaced by alternative biometric characteristics such as voice, as sometimes this is the only feature available. The present study benefits from the advances achieved during last years in understanding and modeling voice production. The paper hypothesizes that a gender-dependent characterization of speakers combined with the use of a set of features derived from the components, resulting from the deconstruction of the voice into its glottal source and vocal tract estimates, will enhance recognition rates when compared to classical approaches. A general description about the main hypothesis and the methodology followed to extract the gender-dependent extended biometric parameters is given. Experimental validation is carried out both on a highly controlled acoustic condition database, and on a mobile phone network recorded under non-controlled acoustic conditions.

## Introduction

Historically, speech signal analysis and processing has attracted wide attention, especially by its multiple applications. For instance, automatic speaker recognition (ASR) or speech synthesis (SS) have been active research areas at least since early 70s (Rosenberg, 1976). More recently, voice has captured again researchers’ attention thanks to its usefulness in order to assess early vocal pathologies, and neurodegenerative and mental disorders among others (Gómez Vilda et al., 2013). Progress achieved thanks to these new applications have allowed for a better understanding of the mechanism of voice production, which have led to an improvement in speaker characterization.

Not only speaker characterization is a key aspect for speaker recognition but it is also a challenging task for different reasons. First of all, voice involves both physiological and behavioral aspects. Regarding physiological characteristics, voices from different individuals differ due to differences in the voice production organs. Regarding behavioral features, voice communication is influenced by socio-cultural and emotional aspects (vocabulary selection, accent, intonation style, etc.). Additionally, voice presents high intra-speaker variability not only due to emotional or temporal health issues, but also due to changes that occur in the voices as a direct result of aging or neurological deterioration.

From a biological point of view, like in any other important motor activity, speech communication requires the interaction of neurophysiologic systems, the motor system, and the sensory system. The motor system plays an important role in speech production, particularly, the vocal tract which can be roughly divided into three different areas: infraglottic, glottal (where the vocal folds are located) and supraglottic, depending on the location or functionality of the different organs and muscles [a comprehensive review can be found in Raphael et al. (2006)].

In voiced speech production, i.e., when vocal fold vibration exists, the joint effects of the subglottal and supraglottal air pressure difference, the laryngeal muscle tension and the elasticity of the vocal folds, cause an opening and closure of the vocal folds, which produces a glottal source. The glottal source can be defined as the sound pressure pattern that is produced in the supraglottic cavities immediately after the vocal folds, which is related to the Liljencrants-Fant excitation, in its ideal form (Fant et al., 1985). This glottal source is then modulated by the supraglottic cavities to produce different categories of sounds/phonetic classes. Therefore, the vocal tract, which includes the speech production organs above the vocal folds, can be regarded as a filter that alters the frequency content of the glottal source due to its resonances (also known as formants: energy amplification) and antiresonances (energy attenuation). This circumstance allows the estimation of the vocal tract shape from the spectral shape of the voice signal (Campbell, 1997).

The different categories of speech in which voiced and unvoiced sounds are classified depend on the manner and point of articulation, which are characterized by the supraglottic cavities. It must be noted that whereas the point and manner of articulation is liable to be imitated, the glottal source, as is linked to vocal fold vibration pattern, is almost impossible to be forged.

Speaker recognition systems have historically used different features in order to cover the variability present in voice (Mazaira Fernández, 2014). Taking into account the different nature of the features use for speaker recognition, we can classify feature extraction modules in two categories: high level features and low-level features.

High level features reflects behavioral characteristics of speakers, such as prosody (pitch, duration, and energy) (Sönmez et al., 1998; Adami et al., 2003; Barra et al., 2006), phonetic information (Kohler et al., 2001; Campbell et al., 2003; Hatch et al., 2005; El Hannani and Petrovska-Delacrétaz, 2007), pronunciation, emotion, stress, idiolect word usage (Doddington, 2001; Boakye and Peskin, 2004), conversational patterns, or other acoustic events (Scheffer and Bonastre, 2005). These differences in the speaking habits result from the manner in which people have learned to use their speech mechanism; but at the same time, the sociolinguistic context, the education and the socio-economic environment play an important role in these differences. The main drawback, as reported in different studies of this kind of systems (Doddington, 1985; Campbell et al., 2004, 2007), is its necessity for more information for both training and testing phases if compared to low-level feature systems and are also easily forged. Another problem is that speaker recognition results tend to be corrupted by the errors in detecting phonetic events. On the other hand, these high-level features are less sensitive to noise and channel mismatch than the low-level ones.

Biometric and spectral levels can be considered as low-level features. Biometric level refers to the use of specific characteristics in the speaker’s production of voice difficult to impost as they are related to physiological or/and behavioral aspects. Among these features we can cite: short-term perturbation in the fundamental frequency (jitter) (Jankowski et al., 1995), perturbations on the cycle-to-cycle phonation amplitude (shimmer) (Farrús et al., 2007). On the other hand, spectral level has been extensively used in speaker recognition systems for feature extraction. Typical methods in this spectral level are as follows: *Short-time spectrum* (no matter if we use the exact representation or its approximation by filter banks) (Xiang and Berger, 2003; Seddik et al., 2004; Burget et al., 2007), *predictor coefficients* [based on a linear model of speech production: (Park et al., 2006)], *formant frequencies and bandwidth* [defined as the resonance frequencies of the vocal tract: (Fatima et al., 2004)], or even the *formant trajectories* (Tanabian et al., 2005).

However, when it comes to practical and real-life scenarios, mel-frequency cepstral coefficients (MFCC) extracted from the power spectral density of speech as a whole seem to have become the *de facto* standard in the area [as demonstrated by its use in almost every system submitted to the 2013 Speaker Recognition Evaluation (SRE) in Mobile Environment (Khoury et al., 2013)]. MFCC templates are usually augmented with dynamic information, using the ΔMFCC (delta MFCC) and ΔΔMFCC (double-delta MFCC) which are polynomial approximations of the first and second derivatives, that give an estimation of how these MFCC features vary over the time. These feature vectors are aligned in streams to build templates for classification purposes. One of the drawbacks of this low-level feature approach is that they fail to capture long-term habitudes in a speaker’s style, such as duration and pausing patterns, intonation, and the use of specific words or phrases. Moreover, the performance of these systems is also affected by acoustic environment variations, noisy channels, and microphone degradation.

Another key aspect that must be considered is the fact that state-of-the-art speaker recognition systems using spectral level parameters do not report to use gender-dependent parameterization neither on the number of MFCC parameters nor on the variables involved on the MFCC extraction process, for instance the number of filters used to compute them.

Moreover, the front-end of a speaker recognition system seems to have been relegated to a secondary plane when compared to the research interest in classification and normalization methods. This actually means that speaker recognition systems usually use the same configuration regardless of the scenario, typically not paying much attention to the front-end subsystem and reporting the use of MFCC in the range of 15–24 computed using between 24 and 32 filters (Kinnunen and Li, 2010; Khoury et al., 2013; Mazaira Fernández, 2014).

As a result, improvements in speaker recognition have been tightly linked to improvements in classification and normalization methods (Kinnunen and Li, 2010) rather than in the use of more accurate parameters to precisely model a speaker.

Nevertheless, when it comes to speaker recognition, the use of the more advanced and accurate classification and normalization systems may not have the expected result if they are not fed with the appropriate features that precisely characterize each speaker. Following this line, the aim of this research is to verify that accurate speaker models can be obtained when gender-dependent parameterization is applied not only to state-of-the-art MFCC features, but also to what we called extended biometric features. These gender-dependent extended biometric (GDEB) features group together parameters extracted from voice-source and tract components, as well as other relevant parameters such as voice formant information. The hypotheses sustaining the proposed methodology are the following: male and female voices exhibit not only acoustic-phonetic differences but physiological variations as well (Whiteside, 2001), therefore a gender-dependent parameterization which also takes advantage of the voice production model, by incorporating voice-source and tract features, will provide a more precise characterization of speakers that will help us to increase overall recognition rates of speaker recognition systems. According to a simplified speech production model, voice can be regarded as the result of filtering an excitation signal with the transfer function of the vocal tract and the lip-radiation model. The methodology followed in this study relies on model inversion (Gómez Vilda et al., 2009) to obtain the following voicing speech estimates: glottal source (more related to phonation/physiology) and vocal tract (more related with acoustics/behavior). This methodology has been adapted to properly work on running speech, solving problems found on early works. Information extracted from these estimates in a gender-dependent basis may be used as a complement to classical parameters in classifying speaker patterns.

The paper is organized as follows: after a brief introduction to voice production, the voice deconstruction algorithm based on inverse filtering and adapted to work on running speech is presented. “Feature Vector Composition” focuses on the frequency-domain parameterization performed on the glottal source estimate (GSE) and vocal tract estimate (VTE) obtained following this methodology. Section “Speaker Recognition” describes the experimental framework defined to validate the proposed GDEB parameterization from different points of view as follows: the speaker recognition system implemented (including not only the front-end but the classification method), the different defined scenarios in which the proposed is evaluated, and the metrics used to measure the performance of the system. The proposed scenarios involved a highly controlled acoustic condition database, and a mobile phone network recorded under non-controlled acoustic conditions database. The results achieved and some comments on these results are presented in Section “Results.” Finally, implications of the study are described in Section “Discussion.”

## Materials and Methods

### Voice deconstruction

As we have already established, both glottal source and vocal tract systems are involved in speech production processes. It may be expected that glottal information will be more influenced by the speaker’s phonation habits, while the description of the vocal tract will be more conditioned by the phonetic structure of the message. On its turn, the power spectral density of the glottal source is strongly conditioned by the biomechanics of the vocal folds. Thus, both vocal tract and glottal information seem to be relevant when characterizing a speaker. In order to establish the influence of vocal and glottal information in speaker recognition applications, it would be useful to break down the speech signal in a GSE and a VTE.

Early implementations (Alku, 1992a; Gómez et al., 2004; Akande and Murphy, 2005) used to deconstruct the voice signals required frame-based pitch-synchronous processing of the glottal source by phonation cycles for the precise estimation of the voice components. However, this requirement is difficult to be met with sounds of dynamic nature (consonants and glides) not to mention when facing continuous speech. To solve this problem, the theory of inverse filtering via linear prediction, applied to Fant’s production model has been used for the reconstruction of the GSE and VTE. More specifically, predictive structures based on the Itakura–Saito Partial Correlation algorithm (PARCOR) (Itakura and Saito, 1970) have been conveniently modified in order to model and invert the voice production system, providing a highly efficient algorithmic structure known as paired lattice.

While a comprehensive description can be found in Mazaira Fernández (2014), Figure 1 provides the block diagram of the voice deconstruction algorithm used to separate the vocal tract and glottal estimates of voice from continuous speech. The proposed algorithm not only allows for a simultaneous estimation of both voice components, but also guarantees that they are orthogonal in terms of correlation. A brief description of the main blocks involved in the process is given below:

- Radiation Compensation Block: a first-order prediction lattice has been implemented to compensate lip-radiation effects.
- Inverse Filtering Block: a k-order filtering process is applied to remove the vocal tract information from the radiation-compensated speech. This process can be implemented using a prediction error lattice.
- Joint-Process Estimation Block (JPE): The residual is used as the reference signal in an Adaptive lattice-ladder filter used for joint-process estimation on the radiation-compensated speech *s*_l_(n). Through this process, a GSE and a VTE are extracted which can be considered fully uncorrelated (second-order decoupling).

**Figure 1:**
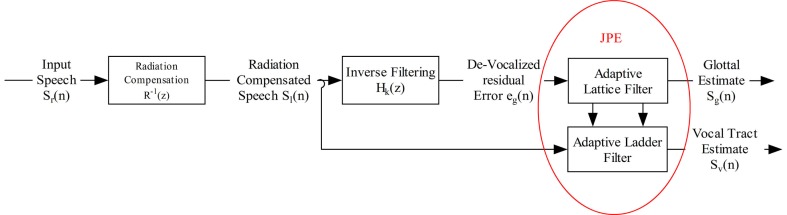
**Separation algorithm using first-order prediction lattice and including a lip-radiation compensation stage**.

**Figure 2:**
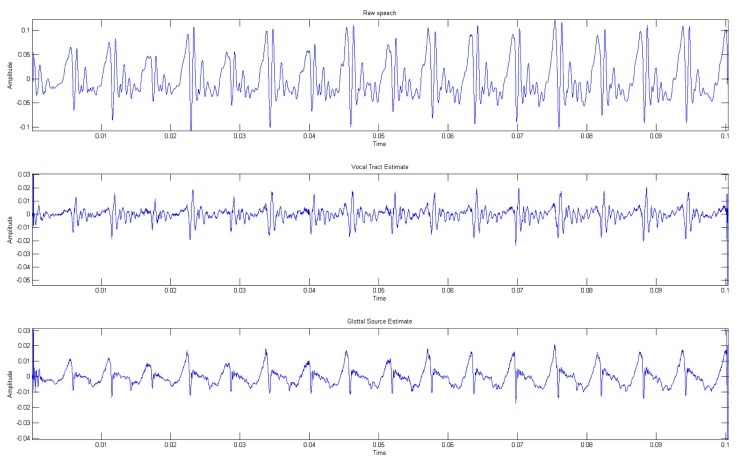
**Vocal tract (middle) and Glottal source (lower) estimates for a female sustained vowel/a/utterance (upper)**.

**Figure 3:**
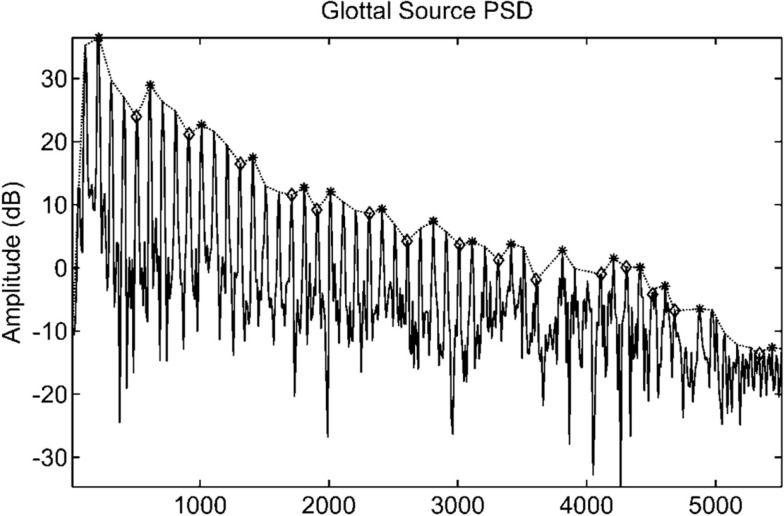
**Power spectral density of the glottal source evaluated over a temporal window which includes multiple glottal cycles**. The relative maxima of the distribution are marked by the harmonics present in the signal. The interconnection of these maxima is known as harmonic envelope or power spectral density profile.

Figure 2 depicts the VTE and GSE obtained from a female speech utterance of vowel/a/applying the described algorithm. Additionally, Figure 3 represents the power spectral density of the GSE, evaluated over a temporal window which includes multiple glottal cycles. This figure clearly shows a peak and trough patterns, agreeing with previous works in the area (Gómez Vilda et al., 2009, 2013; Mazaira Fernández, 2014) that the glottal source do not present a flat spectrum.

The present algorithm needs to be tuned according to the kind of recordings it is supposed to deal with, i.e., we need to find the best parameters in the inverse filtering block that provide the best second-order decoupling in the estimated signals. Different values have been tested for the two specific parameters used in the inverse filtering block, namely the order of the filter and the forgetting factor, which according to Griffiths (1977) “helps it deal better with statistical variations when operating in non-stationary environments.” As the algorithm needs to be tuned accordingly to the kind of recordings, this means that no universal configuration can be provided, instead, when applied, it is necessary to define a development set which collects the variability of data under test and which helps in tuning the meta-parameters of the algorithms used. In other words, the selection of these parameters is done by carrying out a deep search from a number of pre-selected parameters and selecting those for which the recognition system provides better results.

There have been previous attempts to use the glottal source for speaker recognition purposes; however, these approaches differ from our approach not only in the separation algorithm but also in the features derived from the source and tract estimations.

In Backstrom et al. (2002), a method which consists in estimating the vocal tract transfer function using a discrete all-pole (DAP) modeling technique instead of linear prediction coefficients is presented. According to their results, this modification provides better estimation of the first formants, especially the first one, thus decreasing the amount of formant ripple in the estimated glottal flow. However, this improvement is more relevant when applied to high pitch frequency voices and when the vocal tract can be well modeled using an all-pole envelope, which is not always possible.

Previously, Plumpe et al. (1999) introduced a robust approach to identify the glottal closed-phase based, in which the absence of source-filter interaction will result in no or little formant modulation, on first formant tracking calculated from the VTEs. Then the glottal flow results from inverse filtering with a VTE derived from the covariance method within this interval. This method, despite being robust, presents certain drawbacks. First of all, the computational cost derived of using a one-sample shift sliding window for close-phase estimation. It also requires the use of multiple pitch cycles when dealing with high pitch speakers. In addition, the close-phase region detection is clearly affected by the fact that under some circumstances or some voice pathology the vocal folds may never completely close. Finally there is a need for a way to determine a stable and optimal vocal tract function in the close phase. Following the idea of close-phase analysis Akande and Murphy (2005) introduced the Adaptive Estimation of the Vocal Tract transfer function (AEVT) method which is focused more on precise vocal tract estimation rather than on exact source-tract separation. In this adaptive method, the first step consists in removing the glottal frequency by a frequency-selective, multi-pole, zero-phase lag high-pass filter whose role-off is adjusted to meet the low-frequency gain criterion. Using this high-pass filtered data and applying covariance linear prediction analysis and an adaptive algorithm that selects an optimum linear prediction order that satisfies the criteria for minimum phase systems, the vocal tract filter parameters are estimated over a pitch cycle. Finally, removing the effects of the vocal tract and lip radiation from original speech by inverse filtering and subsequent integration provides an estimate of the glottal volume velocity. Again, this method is limited by some problems pointed out by the authors. First, it seems to work only in the cases in which the glottal frequency is lower and sufficiently separated from the first formant which is not always the case. Additionally and probably more important is the fact that although removing the influence of the glottal frequency, the method does not remove glottal contributions over the entire spectrum. Again high pitch voices seem to be problematic.

In Gudnason and Brookes (2008), the Dynamic Programming Projected Phase-Slope Algorithm (DYPSA) (Naylor et al., 2007) to identify glottal closure instants in each cycle is applied. Using multicycle close-phase analysis, an autoregressive model of the vocal tract is estimated and used to produce vocal tract mel-cepstrum coefficients (VTCC). In order to obtain a representation of the voice source (different from glottal source), the VTCC are subtracted from the MFCCs of the speech frame. As already pointed out, accurate detection of closed phase can be difficult under some circumstances such as presence of noise or soft phonation. To overcome this problem, in recent work, Kinnunen and Alku (2009) proposed an approach similar to ours. The iterative adaptive inverse filtering (IAIF) method is applied to extract an estimation of both the vocal tract and glottal source; from this last signal, the source MFCCs are evaluated to capture the frequency-domain characteristics of voice.

The method used in the present paper is related to (but not the same as) the DYPSA algorithm (Kounoudes et al., 2002) or the IAIF algorithm (Alku, 1992b). Both algorithms rely on a simple trick: estimate the vocal tract transfer function independently from the glottal source, either removing a rough estimate of the glottal source from the voiced signal and refining it by iteration (Alku, 1992b), or estimating it during the closed phase of the phonation cycle, when vocal folds are in deep contact (Kounoudes et al., 2002). The underlying reasons availing this *modus operandi* to estimate independently source and filter is very well stated in Gudnason and Brookes (2008): “If we rely on linear prediction modeling of the speech production then we are assuming that the voice source has a flat spectrum and the source becomes encoded in the estimated vocal tract transfer function. To avoid this, we apply closed-phase LPC analysis to circumvent the problem and solve the blind nature of the estimation process.”

In the present work, and this is one of its differential contributions with respect to previous work, we preferred using joint-process estimation of both the vocal tract and the glottal source, under a second-order statistics approach because it formally improves Alku’s method introducing a statistical criterion, and does not need to resource to estimation during the closed phase as in DYPSA, because perfect contact cannot be granted in all circumstances (whispery phonation, modal defective, pathological, female with permanent gap defects, high pitch as in children’s voices, etc.). To our knowledge, this approach has not been used before in source-filter independent estimation.

### Feature vector composition

As previously stated, one of the main objectives of the present study is to probe that a GDEB characterization of speakers can improve the performance of speaker recognition systems. This means that the set of parameters generated by the front-end are going to be different depending on the gender. However, our proposal does not represent a complete break with the classical approach; on the contrary, it can be seen as an extended version where classical MFCC parameters are augmented with MFCC parameters extracted from the VTE and GSE to form the feature vector.

**Figure 4:**
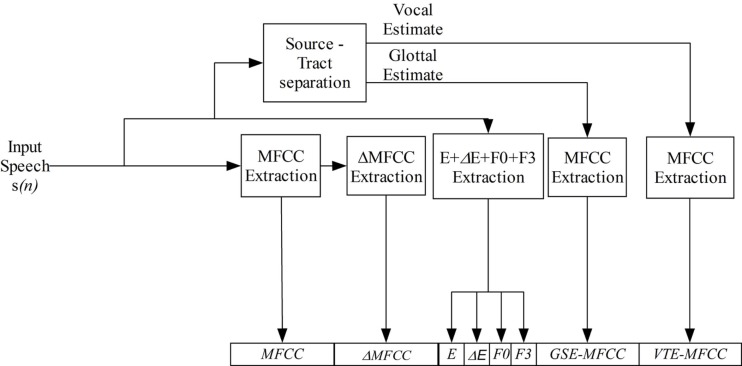
**General parameterization scheme used for both female and male speakers**.

From this point of view, Figure 4 shows the generic form of the feature vector generated by the GDEB front-end; which is common to both genders. Therefore, the differences between male and female feature vectors, rely on the setup of the previously described algorithm (the order of the filter and the forgetting factor coefficient), as well as on the number of filters used to extract the MFCC for the three different signals (raw voice, GSE, and VTE) and the number of MFCC coefficients extracted for each signal. The use of a frequency-domain parameterization of the glottal and VTEs is justified not only for its easy and fast integration into the front-end subsystem, but also for its limited computational impact on overall system.

As it is shown in Figure 4, we have also integrated other parameters frequently used in speaker recognition systems, such as frame energy (E), delta Energy (ΔE), pitch (F0), and a new one such as the third formant (F3) estimate. Energy and ΔE coefficients are typically used as they constitute a heritage from the speech recognition area. ΔE can be defined as the polynomial approximation of the first-order derivative of the Energy. Pitch constitutes a parameter that has been typically used for gender classification purposes as typical average pitch values obtained are 120 Hz for male and 210 Hz for female subjects. However, as it also conveys inter-speaker variability, some works on the speaker recognition area have used this parameter for speaker characterization purposes. In order to estimate the pitch value of speakers, we have developed specific software following the study presented in de Cheveigné and Kawahara (2002).

Finally, we propose the use of F3, which to the author’s knowledge, has not been reported to be specifically used for speaker recognition yet. If we assume that vocal tract length is different among speakers, and taken into account that different size vocal tracts causes the formants to move up and down on the frequency scale, and that formants F1 and F2 are classically associated to vowel detection, we can expect F3 to provide speaker-related information. In order to compute F3, we have followed the approach presented in Welling et al. (2002), in which they applied the use of F3 for speech recognition in the form of vocal tract length normalization.

We must keep in mind that the ultimate goal of this study is to improve speaker recognition systems by incorporating an accurate characterization of speakers. Additionally, the SR system is supposed to operate in different scenarios, i.e., using different databases which include recordings registered under different noise conditions. From this point of view, it is necessary to introduce a noise reduction pre-processing step. In this case, a variation of the Ephraim–Malah spectral subtraction algorithm in a single channel is applied (Ephraim and Malah, 1985). Moreover, we also incorporate the use of typical solutions for SR. Specifically, as depicted in Figure 4, dynamic information extracted from MFCCs is also computed (also known as delta-coefficients or ΔMFCC – polynomial approximations of the first-order derivatives of the static cepstrum), as well as noise and channel distortion reduction techniques in order to deal with non-controlled acoustic conditions scenarios. For this purpose, once the set of MFCC feature vectors have been computed for the whole speech signal, cepstral mean subtraction (CMS), feature warping (FW), and Rasta filtering algorithms are applied. The CMS algorithm (Furui, 1981) mainly consists in computing the mean of each cepstral coefficient over the length of the current utterance, then the mean value is subtracted from the original cepstral coefficient, thus removing the channel-induced effects as well as any other stationary speech component. The aim of the FW process (Pelecanos and Sridharan, 2001) is to transform the original cepstral coefficients so that they follow a specific target distribution, for instance a normal distribution, over a window of speech frames, typically a 3-s window. It provides a set of features that are supposed to be robust to channel mismatch, additive noise and non-linear effects attributed to handset transducers. In the case of RASTA filtering (Hermansky and Morgan, 1994), it tries to remove the spectral components that change at different rates than the one present in speech, i.e., it tries to remove convolutional and additive noise. Last but not least, an adaptive voice activity detector (VAD) algorithm based on energy detection has been implemented, which also incorporates a built-in heuristics that removes or includes recording fragments based on its duration and its relative location to longer voice segments.

There have been also previous attempts of applying source features in order to improve speaker recognition systems. However, it must be noted that not only the results that will be presented improve sensibly the results shown in previous work in general terms, but it has also to be taken to account that the databases used in our experimental framework are by no means more demanding and/or complete than the ones used in previous studies. For instance, Faundez-Zanuy and Rodriguez-Porcheron (1998) report a relative reduction (RR) in the error rate close to 41% [3.68% Equal Error Rate (EER)] when combining LPC cepstrum and LPC residual cepstrum, but the results are obtained on the small DARPA TIMIT Database (24 male and 14 female speakers). Markov and Nakagawa (1999) also consider the use of pitch and/or LPC residual combined with the main LPC-derived cepstral coefficients, reporting a reduction of the speaker identification rate from 98.5 to 97% using the High quality NTT Database. Zheng et al. (2007) introduce the use of the wavelet octave coefficients of residues (WOCOR), which are generated by applying a pitch-synchronous wavelet transform to the residual signal, to provide complementary information to classical MFCC. They report a reduction on the EER from 9.30% of the conventional MFCC-based system to 7.67%. Tests are run on the male subset of the NIST 2001 database, using training utterances for each speaker of about 2 min long. Gudnason and Brookes (2008) not only provide results achieved using YOHO database (recorded in a normal office environment) and the TIMIT database (high quality and controlled environment) but also points out the fact that “direct comparison of results based on different databases is impossible”. Plumpe et al. (1999) also provide results on a subset of the TIMIT database (NTIMIT – result of transmitting the TIMIT database over the telephone network). In both cases (Plumpe et al., 1999; Gudnason and Brookes, 2008), improvements in the recognition rates are reported when source coefficients are combined with classical parameters, but global recognition rates are still far from accurate as for instance, for the YOHO database the misclassification rate decreased from 13.79 to 10.07%.

More recently, Vandyke et al. (2013) also report some improvements [but global misclassification rates on the same order as in Plumpe et al. (1999) and Gudnason and Brookes (2008)], using the YOHO database, on the identification rates when voice-source features are used in this case using smaller cohort sizes and support vector machines as classifier. Hanilçi and Ertas (2011) uses the more challenging NIST 2001 SRE database, but using as much as 2-min training utterance per speaker, to conclude that glottal flow features convey useful speaker-specific information. However, even in the case of applying fusion of scores of classical and source feature set pairs by linear score weighting, results are far from the NIST SRE state-of-the-art recognition rates. Finally, it is important to notice that none of the cited works report the use of gender-dependent parameterization.

### Speaker recognition

From a practical point of view, progress in speaker characterization has a direct effect in speaker recognition systems. Indeed, an adequate way of verifying that an improvement in characterizing a speaker is achieved is by obtaining an improvement in the recognition rates of the speaker recognition system in which this characterization is used. The following sections are devoted to present the speaker recognition system implemented (including not only the front-end but the classification method), the different scenarios in which the system is evaluated, i.e., the different databases used in the experiments, and finally the metrics used to evaluate the performance of the system.

#### GMM–UBM Speaker Recognition System

**Figure 5:**
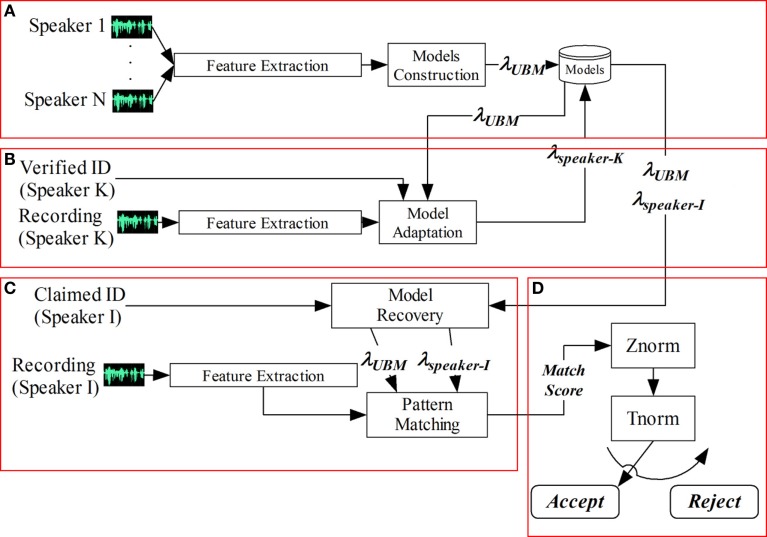
**GMM–UBM speaker verification system**. **(A)** UBM training. **(B)** GMM speaker model building. **(C)** Speaker Verification. **(D)** Score normalization.

Although new modeling strategies have been proposed in recent years in order to improve recognition rates (Kinnunen and Li, 2010; Mazaira Fernández, 2014), the Gaussian mixture model (GMM)–universal background model (UBM) probabilistic paradigm strategy is still considered the *de facto* reference method in text-independent speaker recognition when the available amount of information for training purposes is limited. Figure 5 provides a block diagram of the speaker recognition system implemented applying the GMM–UBM approach. In this section, we do not care about the feature extraction process which has been already presented.

In the set of experiments that has been carried out, we have used a standard mixture classifier with diagonal covariance matrix. Each speaker is represented by a GMM, λ_speaker-k_, which has been adapted from a gender-dependent UBM using the MAP algorithm in which only the distribution means have been adapted (part B in Figure 5). The UBM is also represented as a GMM, λ_UBM_, which has been trained from the training set via the expectation maximization (EM) algorithm (part A in Figure 5). The number of Gaussians, as well as the relevance factor used in the MAP algorithm, depends on the specific experiment carried out.

The log-likelihood ratio (LLR) has been the score used to take a decision on whether a test audio-segment is likely to be spoken by a specific speaker, with claimed identity λ_speaker-i_. In other words, the set of feature vectors extracted from a test audio-segment is compared with the claimed speaker model giving a match score which measures their similarity (part C in Figure 5).

(1) $$\text{LLR}\left(X,\lambda_{\text{speaker-i}}\right)=\log\;P\left(X|\lambda_{\text{speaker-i}}\right)-\log\;P\left(X|\lambda_{\text{UBM}}\right)$$

Additionally, the decision scores can be normalized using zero normalization [ZNorm – (Li and Porter, 1988)] and test normalization [TNorm – (Auckenthaler et al., 2000)] or by combining both of them. In the case of ZNorm, the normalized score is given by:

(2) $$S_{\text{ZNorm}}=\frac{\log\left(P\left(X|\lambda_{\text{speaker-i}}\right)\right)-\mu_{\text{Z}}}{\sigma_{\text{Z}}}$$

where μ*_z_* and σ_z_ are the mean and standard deviation for the impostor distribution. To estimate these values, the target speaker model is tested against utterances from impostors; this results in a set of likelihood scores from which the impostor distribution is estimated.

TNorm also uses mean and standard deviation as normalization parameters, but in this case a set of impostors is used to estimate the log-likelihood for the test input utterance. So mean and variance are computed for these impostor scores and the normalized score is computed like in previous equation. It must be noted that in the set of experiments defined using the ALBAYZIN and MOBIO databases, the number of available speakers for normalization purposes is quite limited. So the results obtained when applying these normalizations will be influenced by this fact.

Finally, a decision is made to either accept or reject the claimant according to the match scores, and a specific threshold (part D in Figure 5).

### Test framework

The main objective of the speaker recognition is to determine whether a specific target is present or not in a given speech segment. We have defined two different scenarios which involve the use of two different databases: ALBAYZIN (Casacuberta et al., 1991) and MOBIO (McCool et al., 2012) databases. The ALBAYZIN database allows us to define highly controlled acoustic scenario without channel variability, with gender and age variability. The use of the MOBIO speech corpus constitutes and additional challenge as it contains text-independent recordings acquired in non-controlled mobile environments. Additionally, as ALBAYZIN is a Spanish database and MOBIO is an English recorded database, we are testing our system against language variability.

In each of the two proposed scenarios, the databases are split into three different subsets as follows:

- Background training set: used to learn the background parameters of the algorithm (UBM, subspaces, etc.) or for normalization purposes.
- Development set: the data assigned to this set are split into two subsets: enrollment and test. The first one is used to create a model of each of the target speakers. The second one contains a list of audio samples that must be tested against all the target speakers. The data on this set are supposed to be used to tune meta-parameters of the algorithm (e.g., number of Gaussians, dimension of subspaces, etc.). The recognition rate, regarding EER, achieved with this development set is used to define a score threshold that will be used to evaluate the performance of the recognition systems.
- Evaluation set: the final evaluation performance is analyzed using this set, which has a similar structure as the development set. A score must be provided for each trial, for instance in the form of log-likelihood, representing how accurately the test segment is classified as containing, or not, speech for the target speaker against which is confronted. It must be noted that the setup established in the development set (regarding not only meta-parameters but also speaker characterization) is going to be the one used in this set.

It must be noted that no cross-gender trials are going to be present in the tests. According to state-of-the-art in speaker recognition, gender detection is taken for granted as deduced from the fact that none of the most important SREs, NIST (http://www.itl.nist.gov/iad/mig/tests/spk/) or SRE in Mobile Environments (Khoury et al., 2013), incorporates cross-gender trials. Table 1 summarizes the amount of data included in each of the subsets, in terms of number of recordings, number of target speakers, and the total amount of trials for the ALBAYZIN scenario and for MOBIO database. In the case of ALBAYZIN each recording contains 4 s of speech on average, whereas in the case of MOBIO, each recording last between 2 and 10 s.

**Table 1:** **Description of the contents of the different subsets for the different scenarios**.

	MOBIO					ALBAYZIN				
	Background					Background				
	Enrollment					Enrollment		
	Speakers	#Files				Speakers	#Files			
**MALE**	37	7104				25	75			
**FEMALE**	13	2496				25	75			
**TOTAL**	50	9600				50	150			

	**Development**					**Development**				
	**Enrollment**		**Test**			**Enrollment**		**Test**		
	**#Targets**	**#Files**	**Speakers**	**#Files**	**#Trials**	**#Targets**	**#Files**	**Speakers**	**#Files**	**#Trials**

**MALE**	24	120	24	2520	60480	25	75	25	550	13750
**FEMALE**	18	90	18	1890	34020	25	75	25	550	13750
**TOTAL**	42	210	42	4410	94500	50	150	50	1100	27500

	**Evaluation**					**Evaluation**				
	**Enrollment**		**Test**			**Enrollment**		**Test**		
	**#Targets**	**#Files**	**Speakers**	**#Files**	**#Trials**	**#Targets**	**#Files**	**Speakers**	**#Files**	**#Trials**

**MALE**	38	190	38	3990	151620	88	264	88	4136	363968
**FEMALE**	20	100	20	2100	42000	88	264	88	4136	363968
**TOTAL**	58	290	58	6090	193620	176	528	176	8272	727936

### Metrics

In order to evaluate the performance of the systems, regardless the scenario, we will use the EER quality measure, and the Half Total Error Rate (HTER) which can be defined from a score threshold, θ_dev_, obtained from the development set as follows:

(3) $$\theta_{\text{dev}}{=}_{\;\theta }^{\arg\;\min}|\text{FA}{\text{R}}_{\text{dev}}\;\left(\theta \right)-\text{FR}{\text{R}}_{\text{dev}}\;\left(\theta \right)|$$

where FAR is the false acceptance rate and FRR is the false rejection rate. This score threshold, θ_dev_, provides the EER operating point for the system on development:

(4) $$\text{EER}=\frac{\text{FA}{\text{R}}_{\text{dev}}\left(\theta_{\text{dev}}\right)+\text{FR}{\text{R}}_{\text{dev}}\left(\theta_{\text{dev}}\right)}{2}$$

This threshold is then used on the evaluation data set to obtain the HTER that can be defined as:

(5) $$\text{HTER}=\frac{\text{FA}{\text{R}}_{\text{eval}}\left(\theta_{\text{dev}}\right)+\text{FR}{\text{R}}_{\text{eval}}\left(\theta_{\text{dev}}\right)}{2}$$

However, as no cross-gender trials are going to be present in the tests, we can define a new metric which we call Half Equal Error Rate (HEER):

(6) $$\text{HEER}=\frac{\text{EE}{\text{R}}_{\text{M}}+\text{EE}{\text{R}}_{\text{F}}}{2}$$

As it must be noted, we distinguish between EER for male (EER_M_) and female (EER_F_) speakers. Obviously, when using a gender-independent parameterization, it is necessary to reach a compromise between both EER in order to minimize HEER. However, when using a gender-dependent parameterization, this compromise disappears and the objective is to minimize EER_M_ and EER_F_ independently. It is worth noting that the threshold used will be different depending on the gender, since no normalization has been carried out on the scores in order to obtain a universal threshold.

Additionally, the error rates are going to be represented using the detection error trade-off (DET) curves, where the FRR is plotted against the FAR. The DET curves can be used to evaluate the calibration of the verification system.

Finally, through this paper, two tailed *p*-values are obtained by means of *Z*-test following the procedure proposed in Chu Wu et al. (2013) to solve test data dependencies due to multiple uses of the same subjects. A detection cost function defined as a weighted sum of the probabilities of type I error (miss) and type II error (false alarm) is employed as a metric. The detection cost function may be approximately normally distributed regardless of the distributions of target scores and non-target scores under the assumption that the distribution of 2000 bootstrap replications of the statistic of interest is normal. The standard error (SE) of the detection cost function is estimated using a two-layer non-parametric two-sample bootstrap method.

## Results and Discussion

### Baseline front-end

In order to evaluate the influence of what we have called GDEB, an additional system, called baseline front-end also connected to the same modeling and scoring system was developed. This baseline front-end performs a classical feature extraction, providing gender-independent speaker features based on MFCC + Δ, extracted using the same setup for both genders (see Figure 4).

### Text-independent speaker recognition under controlled conditions (ALBAYZIN)

#### A Classical MFCC Parameters Configuration

In order to find the best configuration, i.e., the one which minimizes the function HEER, a battery of tests has been conducted on the development set using the baseline front-end in a gender-independent configuration (GIC). As we are using the GMM–UBM approach, we can configure the number of MFCC, the number of filters (*F*) used to obtain the MFCC, the number of Gaussians (*G*) used in the model, as well as the relevance factor in the MAP adaptation algorithm (α). Additionally, we have also evaluated the usefulness of the double delta-coefficients, as they are state-of-the-art features in speaker recognition systems.

If we establish a gender-dependent configuration of the system, then we can minimize independently the EER for each gender. In this case, for instance, the number of filters used to compute MFCC as well as the MAP relevance factor and the Gaussians in each model are different for each gender. According to the results shown in Table 2, GIC provides slightly worse results than the ones obtained using a gender-dependent characterization (GDC) even in the case in which we are using just MFCC coefficients extracted from the power spectral density of speech as whole. Moreover, the use of MFCCs + Δ + ΔΔ configuration does not offer additional benefits neither for male nor for female speakers.

**Table 2:** **Configurations providing most successful results in terms of EER for GDC and GIC for the ALBAYZIN development set scenario [RR ***→*** Relative Reduction/[threshold]/(*p*-value)]**.

Parameters	Genre	*G*	*α*	*F*	*MFCC*	EER_M_ [*θ*_M_] (*p*-value)	EER_M_ RR	EER_F_ [*θ*_F_] (*p*-value)	EER_F_ RR	HEER [RR]
Gender-independent configuration (GIC MFCCs + Δ)	M/F	256	5	34	26	2.534% [−0.178]	–	2.170% [−0.169]	–	2.352% [–]
Gender-independent configuration (GIC MFCCs + Δ + ΔΔ)	M/F	256	5	50	26	3.042% [−0.401] (2.04 × 10^−1^)	−20.05%	2.409% [−0.375] (3.06 × 10^−1^)	−11.01%	2.725% [−15.85%]
Gender-dependent configuration (GDC MFCCs + Δ)	M	256	16	34	26	12.390% [−0.001] (5.57 × 10^−1^)	5.68%			2.193% [6.76%]
	F	256	5	44	26			1.996% [−0.166] (4.07 × 10^−1^)	8.02%		

*The results highlighted in green, are the ones achieving higher recognition rates*.

#### Gender-Dependent Extended Biometric Configuration

Once we have established a baseline to be beaten, and that we have verified that a gender-dependent characterization provides a clear advantage for speaker recognition purposes, we continue the experiments by introducing what we call alternative parameters into the best GDC, as is the one providing more accurate recognition results. We have tested all possible combinations of these parameters (i.e., Energy, ΔEnergy, Pitch, and F3); however, Table 3 just reflects the configuration providing better results in terms of EER. As we are dealing with good quality recordings without channel variability, the use of Energy and ΔEnergy features combined with classical MFCC for both male and female speakers provides an important improvement in the recognition rates. Additionally, F0 and F3 also take part in the configuration providing the most successful results in terms of EER for female speakers.

**Table 3:** **EER_M_, EER_F_, and HEER obtained on development set (no score normalization), comparing classical parameters with extra parameters and extended biometric parameters for the configurations providing the most successful results [RR ***→*** Relative Reduction/[threshold]/(*p*-value)]**.

Parameters	Genre	GSE *+* VTE setup	Extra parameters	EER_M_ [*θ*_M_] (*p*-value)	EER_M_ RR	EER_F_ [*θ*_F_] (*p*-value)	EER_F_ RR	HEER [RR]
Gender-independent configuration (GIC MFCCs + Δ)	M/F	–	–	2.534% [−0.178]	–	2.170% [–0.169]	–	2.352% [–]

Gender-dependent configuration (GDC MFCCs + Δ)	M	–	–	2.390% [−0.001] (5.57 × 10^−1^)	5.68%			2.193% [6.76%]

	F	–	–			1.996% [−0.166] (4.07 × 10^−1^)	8.02%	

Gender-dependent configuration (GDC MFCCs + Δ + Extra)	M	–	E + ΔE	2.163% [−0.035] (2.09 × 10^−1^)	14.64%			1.991% [15.37%]

	F	–	E + ΔE + F0 + F3			1.818% [−0.113] (2.12 × 10^−1^)	16.23%	

Gender-dependent configuration (GDC MFCCs + Δ + Extra + GSE)	M	**Source-tract sep**. **Alg** Prediction order: 10 Forgetting factor: 0.995 **GSE** 13-Channel Filter bank 8 MFCC	E + ΔE	1.504% [−0.131] (5.41 × 10^−4^)	40.65%			1.477% [37.19%]

	F	**Source-tract sep**. **Alg** Prediction order:16 Forgetting factor: 0.995 **GSE** 12-Channel Filter bank 4 MFCC	E + ΔE + F0 + F3			1.451% [−0.145] (1.67 × 10^−3^)	33.15%	

*The results highlighted in green, are the ones achieving higher recognition rates*.

Next, we verify the viability of using the extended biometric parameters extracted by the GDEB front-end, for speaker recognition purposes in the text-independent scenario. The approach that has been followed consists in incorporating the extended biometric coefficients (information extracted from the VTE and GSE) into the best gender-dependent configuration selected so far, in two stages. First, we incorporate a set of parameters extracted from the GSE, and once a specific configuration improving previous results is found, we continue by incorporating parameters extracted from the VTE. We proceed this way because the VTE is more related to the message carried out by voice, rather than to the biometry of the speakers; therefore as we are dealing with text-independent trials, GSE is supposed to provide more benefits than VTE in terms of recognition rates.

Although multiple configurations have been tested, regarding the multiple variables that can be tuned in the GDEB front-end, Table 3 shows the ultimate configurations chosen for each gender, as well as the recognition rates obtained in each case in terms of EER_M_, EER_F_, and HEER. Additionally, the RR in terms of EER_X_ and HEER compared to the GIC MFCCs + Δ configuration is also provided. It must be noted that a RR of 40% in terms of EER_M_ is achieved with respect to the GIC in the case of male speakers, while a RR of more than 30% in terms of EER_F_ is achieved in the case of female speakers.

**Figure 6:**
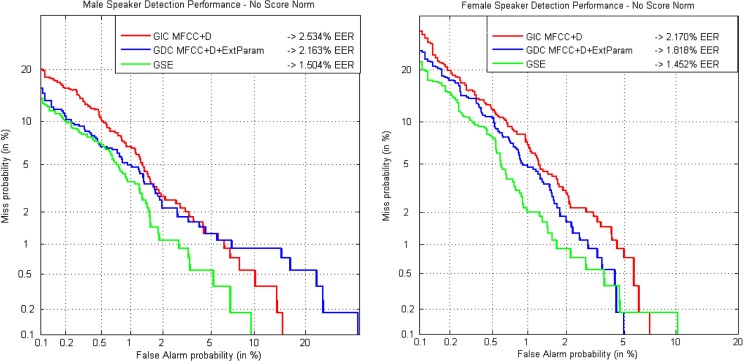
**DET curves comparing classical parameters in a gender-independent setup with the GDEB parameterization on ALBAYZIN development set for male (left) and female (right) speakers**.

DET curves corresponding to the results presented in Table 3 are depicted in Figure 6 for both male and female speakers. Clearly, the parameterization generated by the GDEB front-end, in this case just including information from the GSE in the form of MFCC, is the one providing the best results on the development set for male and female speakers. It must be reminded that the goal of the test is the reduction of EER and not the area under the curve (AUC), thus it may happen that GIC shows better results than GDC for some of the points of the curve. However, GDC will always produce better or at least equal results than GIC in terms of EER.

**Figure 7:**
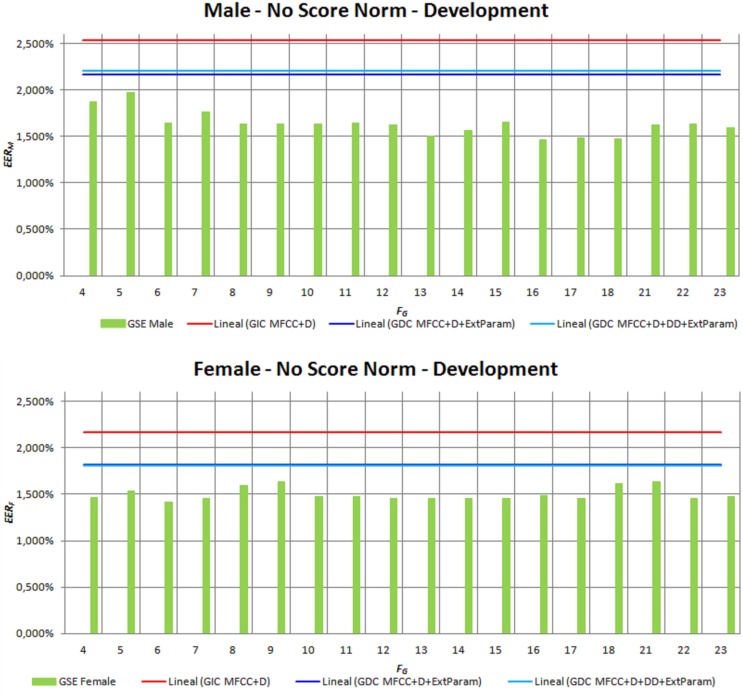
**Influence of the GSE configuration on the results achieved on terms of EER for both male and female speakers on development set**.

We have also verified that the improvement derived from incorporating GSE information into the feature vector is systematically obtained and is not the result of an isolated and specific configuration. Figure 7 (upper) provides in green solid line the minimum EER_X_ (*y*-axis) obtained when GSE is incorporated into the feature vector in the form of MFCCs for male speakers. Different numbers of MFCC*_G_* = {2,4,6,8,10} have been tested, which have been computed applying a filter bank with different numbers of filters *F_G_* = [4…23] (*x*-axis). Each point in the *x*-axis represents the minimum EER obtained for a specific value of *F_G_*, regardless the MFCC_G_ values. Figure 7 (lower) provides the same information for female speakers. Clearly, from the depicted results, the use of GSE systematically results in an improvement of recognition rates regardless the gender of speakers.

Another factor that must be analyzed is the influence of score normalization algorithms in recognition rates. For this reason, the same experiments that have been presented so far (when no score normalization is applied) have been conducted but applying ZNorm, TNorm, and ZTNorm. From the results achieved (see Table 4), it is clear that no matter whether score normalization is applied or not, the best results in terms of EER_X_ or HEER are always obtained when GSE information is incorporated on the feature vectors. The use of score normalization algorithms provides a general improvement on all recognition rates. Specifically, TNorm provides the best results in terms of EER_F_ while ZTNorm provides the best results in terms of EER_M_. However, the results achieved using these normalization algorithms are going to be influenced by the quality and amount of available data for this purpose.

**Table 4:** **HTER_X_ on evaluation set, obtained for configurations providing the most successful results on development set, applying different score normalizations [RR ***→*** Relative Reduction/[threshold]/(*p*-value)]**.

Score norm	Parameters	EER_M_ [***θ***_M_] (*p*-value)	HTER_M_ (*p*-value)	HTER_M_ RR	EER_F_ [***θ***_F_] (*p*-value)	HTER_F_ (*p*-value)	HTER_F_ RR
**NO Norm**	Gender-Independent configuration (GIC MFCCs + Δ)	2.534% [−0.178]	3.347%	–	2.170% [−0.169]	3.250%	–
	Gender-dependent configuration (GDC MFCCs + Δ + Extra)	2.163% [−0.035] (2.09 × 10^−1^)	3.089% (3.86 × 10^−1^)	7.70%	1.818% [−0.113] (2.11 × 10^−1^)	3.094% (8.51 × 10^−1^)	4.79%
	Gender-dependent configuration (GDC MFCCs + Δ + Extra + GSE)	1.504% [−0.131] (5.40 × 10^−4^)	2.189% (6.37 × 10^−13^)	34.61%	1.451% [−0.145] (1.67 × 10^−3^)	2.673% (2.97 × 10^−2^)	17.74%
**ZTNorm**	Gender-independent configuration (GIC MFCCs + Δ)	2.000% [1.847]	2.783%	–	1.655% [2.233]	3.081%	–
	Gender-dependent configuration (GDC MFCCs + Δ + Extra)	1.636% [1.995] (6.32 × 10^−2^)	2.432% (8.60 × 10^−7^)	12.59%	1.455% [2.305] (1.73 × 10^−1^)	2.870% (3.97 × 10^−1^)	6.85%
	Gender-dependent configuration (GDC MFCCs + Δ + Extra + GSE)	1.273% [2.031] (1.30 × 10^−3^)	1.977% (0.00)	28.94%	1.273% [2.304] (2.41 × 10^−2^)	2.709% (4.84 × 10^−1^)	12.07%
	Gender-dependent configuration (GDC MFCCs + Δ + Extra + GSE + VTE)	1.114% [2.092] (3.21 × 10^−4^)	1.917% (1.11 × 10^−15^)	31.12%	–	–	–
**TNorm**	Gender-independent configuration (GIC MFCCs + Δ)	2.000% [1.004]	2.806%	–	1.455% [1.118]	2.835%	–
	Gender-dependent configuration (GDC MFCCs + Δ + Extra)	1.807% [1.199] (3.96 × 10^−1^)	2.555% (3.14 × 10^−4^)	8.93%	1.424% [1.238] (8.20 × 10^−1^)	2.598% (1.61 × 10^−1^)	8.36%
	Gender-dependent configuration (GDC MFCCs + Δ + Extra + GSE)	1.288% [1.252] (8.70 × 10^−4^)	1.812% (2.22 × 10^−16^)	35.42%	1.133% [1.151] (1.84 × 10^−4^)	2.289% (1.15 × 10^−1^)	19.28%
	Gender-dependent configuration (GDC MFCCs + Δ + Extra + GSE + VTE)	–	–	–	1.091% [1.270] (7.48 × 10^−2^)	2.262% (2.29 × 10^−1^)	20.21%
**ZNorm**	Gender-independent configuration (GIC MFCCs + Δ)	2.045% [2.477]	3.388%	–	1.818% [2.886]	3.075%	–
	Gender-dependent configuration (GDC MFCCs + Δ + Extra)	1.848% [2.794] (6.30 × 10^−2^)	3.040% (2.49 × 10^−3^)	10.25%	1.655% [3.228] (4.50 × 10^−1^)	3.203% (4.54 × 10^−2^)	−4.16%
	Gender-dependent configuration (GDC MFCCs + Δ + Extra + GSE)	1.496% [2.777] (1.25 × 10^−3^)	1.980% (0.00)	41.55%	1.231% [3.030] (1.88 × 10^−3^)	2.635% (2.95 × 10^−2^)	14.31%

*The results highlighted in green, are the ones achieving higher recognition rates*.

To complete the study, we have to verify the usefulness of the information provided by the VTE. However, we have limited the test to the most successful male and female configurations in terms of EER_M_ and EER_F_, i.e., GSE ZTNorm for male speakers and GSE TNorm for female speakers. Table 4 provides the results obtained on the evaluation set using the different configuration previously selected for different score normalization techniques, and the selected threshold.

**Figure 8:**
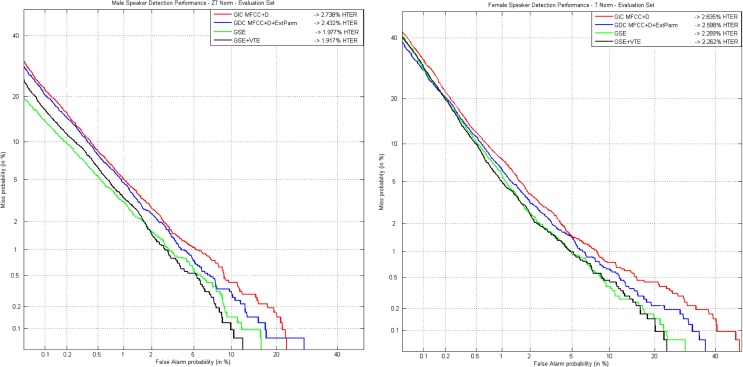
**DET curves comparing classical parameters in a gender-independent setup with the GDEB parameterization on ALBAYZIN evaluation set for male (left) and female (right) speakers**.

From the results depicted in Table 4, it is clear that relevant improvements achieved on development set when using the GDEB front-end remain consistent with the evaluation set, where the system is exposed to new and unknown data. Figure 8 shows the results achieved for male (left) and female (right) speakers when ZTNorm and TNorm are, respectively, applied.

### Text-independent speaker recognition in mobile environments (MOBIO)

#### A Classical MFCC Parameters Configuration

In the case of the scenario described using the MOBIO database, we proceed in a similar way. We start by finding a configuration using the baseline front-end in a gender-independent setup which provides the most accurate results in terms of HEER. In other words, we run several experiments on the development set fixing different values for the different variables that can be tuned: the number of MFCC, the number of filters (*F*) used to obtain the MFFC, the number of Gaussians (*G*) used in the model, as well as the relevance factor (α). The selected configuration is labeled as GIC. As previously said if we use a gender-dependent characterization, we can minimize each gender EER independently, which actually means selecting different values for each of the parameters depending on the gender. Table 5 provides the results achieved on the development set for this two approaches GIC and GDC, when no score normalization is applied. Yet again the use of a gender-dependent setup using classical MFCC parameters proves its value in order to improve recognition rates of a speaker recognition system.

**Table 5:** **Configurations providing most successful results in terms of EER for GDC and GIC for the MOBIO development set scenario [RR → Relative Reduction/[threshold]/(*p*-value)]**.

Parameters	Genre	*G*	***α***	*F*	MFCC	EER_M_ [***θ***_M_] (*p*-value)	EER_M_ RR	EER_F_ [***θ***_F_] (*p*-value)	EER_F_ RR	HEER [RR]
Gender-independent configuration (GIC MFCCs + Δ)	M/F	256	24	30	27	11.567% [−0.007]	–	11.693% [−0.009]	–	11.630% [–]
Gender-dependent configuration (GDC MFCCs + Δ)	M	1024	24	44	25	10.654% [0.014] (9.81 × 10^−2^)	7.89%			11.150% [4.12%]
	F	256	24	34	24			11.646% [0.011] (8.49 × 10^−1^)	0.40%	

*The results highlighted in green, are the ones achieving higher recognition rates*.

#### Gender-Dependent Extended Biometric Configuration

**Figure 9:**
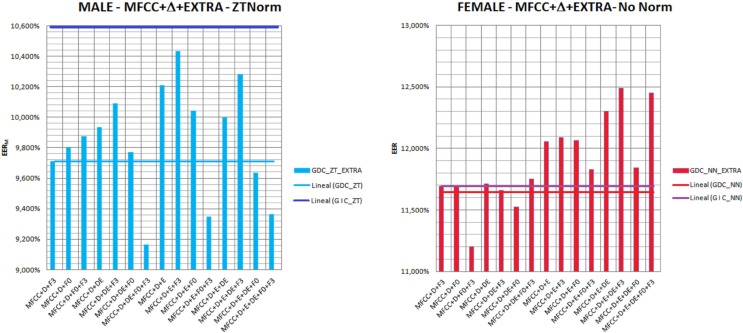
**EER achieved for male and female speakers, when ZTNorm in the case of male (left) and NoNorm in the case of female (right) speakers are applied, in a gender-dependent setup which incorporates different combinations of extra parameters**.

We continue the test by introducing what we call alternative parameters into the best GDC, as is the one providing more accurate recognition results. We have tested all possible combinations of these parameters. Additionally, we also analyzed the effect of score normalization techniques, i.e., ZTNorm, ZNorm, TNorm, and NoNorm (which means that no score normalization technique is applied). Figure 9 provides the results achieved for male and female speakers, when ZTNorm in the case of male (left) and NoNorm in the case of female (right) speakers are used. The selected normalization techniques are justified taking into account the best results achieved in terms of EER. In the case of female speakers, the application of any kind of normalization will worsen the results as the amount of data available for normalization purposes is quite limited. Table 6 provides the most successful results achieved in terms of EER on the development set. In this scenario where the quality of the recordings regarding background noise are quite poor, the use of parameters F0 and F3 are more relevant for speaker characterization than E or ΔE.

**Table 6:** **EER obtained on development set (ZTNorm – male and NoNorm – female), comparing classical parameters in a gender-independent setup with a gender-dependent setup in which extra parameters and extended biometric parameters are incorporated [RR ***→*** Relative Reduction/[threshold]/(*p*-value)]**.

Parameters	Genre	GSE ***+*** VTE setup	Extra parameters	EER_M_ [***θ***_M_] (*p*-value)	EER_M_ RR	EER_F_ [***θ***_F_] (*p*-value)	EER_F_ RR	HEER [RR]
Gender-independent configuration (GIC MFCCs + Δ)	M/F	–	–	10.594% [1.556]	–	11.693% [−0.009]	–	11.143% [–]
Gender-dependent configuration (GDC MFCCs + Δ + Extra)	M	–	ΔE + F0 + F3	9.165% [1.597] (2.02 × 10^−5^)	13.5%			10.183% [8.61%]
	F	–	F0 + F3			11.201% [0.016] (2.36 × 10^−1^)	6.37%	
Gender-dependent configuration (GDC MFCCs + Δ + Extra + GSE)	M	**Source-tract sep**. **Alg** Prediction order: 24 Forgetting factor: 0.995 **GSE** 7-Channel Filter bank 6 MFCC	ΔE + F0 + F3	8.332% [1.619] (1.38 × 10^−8^)	21.3%			9.48% [14.92%]
	F	**Source-tract sep**. **Alg** Prediction order: 36 Forgetting factor: 0.995 **GSE** 22-Channel Filter bank 4 MFCC	F0 + F3			10.643% [0.010] (4.84 × 10^−4^)	8.98%	
Gender-dependent configuration (GDC MFCCs + Δ + Extra + GSE + VTE)	M	**VTE**	ΔE + F0 + F3	8.496% [1.506] (2.09 × 10^−5^)	19.8%			9.75% [12.50%]
		14-Channel	
		Filter bank	
		2 MFCC	
	F	**VTE**	F0 + F3			11.016% [0.023] (2.02 × 10^−1^)	5.79%	
		25-Channel	
		Filter bank						
		2 MFCC						

*The results highlighted in green, are the ones achieving higher recognition rates*.

The next step, like in the previous scenario, consists in introducing what we have called extended biometric parameters extracted by the GDEB front-end. The approach that has been followed, consists in incorporating, the set of parameters extracted from the GSE into the most successful setup, i.e., GDC MFCCs + Δ + ΔE + F0 + F3 in the case of male speakers when ZTNorm is applied and GDC MFCCs + F0 + F3 in the case of female speakers when NoNorm is applied. Table 6 provides the ultimate configuration selected for each gender, as well as the recognition rates obtained in each case in terms of EER_M_, EER_F_, and HEER. Additionally, the RR in terms of EER_X_ and HEER compared to the GIC MFCCs + Δ configuration is also provided.

**Figure 10:**
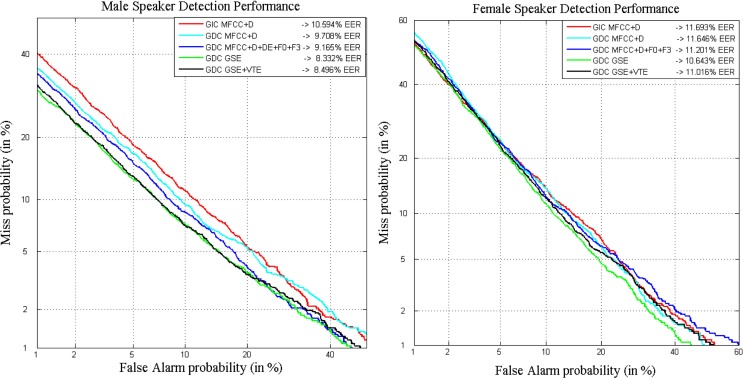
**DET curves comparing classical parameters in a gender-independent setup with the GDEB parameterization on MOBIO development set for male (left) and female (right) speakers**.

The DET curves that represent the results obtained with each of the previously presented configurations are depicted in Figure 10 for male speakers (left) and for female speakers (right). Clearly, the proposed GDEB parameterization, in this case incorporating information just from the GSE in the form of MFCCs, is the configuration that provides the most successful results in the development set for both male and female speakers. The different tests carried out including the VTE parameters are worse than the results obtained using GSE parameters, but still improve recognition rates of GIC, as expected. Specifically, for the male speakers, the use of GSE setup, thus a gender-dependent configuration incorporating extended biometric features, provides a RR of 21% in terms of EER_M_, with respect to the GIC. Whereas in the case of female speakers, the use of the GSE setup allows for a relative reduction close to 11% in terms of EER_F_, with respect to the GIC.

What is more interesting about this scenario is the fact that thanks to the SRE in Mobile Environments (Khoury et al., 2013), we can compare the results achieved by the system proposed in this work with other systems under the same scenario and almost same constraints. Table 7 summarizes the results obtained in the SRE by different systems in both development and evaluation set. Names of the systems correspond to the research groups that have developed the systems according to Khoury et al. (2013). Systems marked with * (also highlighted in red), are actually fusion of different systems, while systems marked with + (also highlighted in orange) are those who used external/additional training data. It must be noted that, despite having developed a simple recognition system (based on the UBM–GMM paradigm), the fact of having achieved a better speaker’s characterization based on gender-dependent biometric parameters allows us to get very competitive results (GIAPSI systems on Table 7). It is important to point out that the only systems that improve the recognition rates of our system are those that either performed a fusion of multiple systems or used additional data for training. Regarding fusion, the first row of Table 7 provides the hypothetical results that a system fusing all systems will achieve, which means that system fusion will usually perform better than stand alone system. Regarding the use of additional data for training, it may have an important effect when using normalization techniques if adequate data are selected.

**Table 7:** **EER on the development set and HTER on the evaluation set for the systems participating in *2013 SRE in Mobile Environments***.

System name	MALE			System name	FEMALE	
	Development set EER (*p*-value)	Evaluation set HTER (*p*-value)			Development set EER (*p*-value)	Evaluation set HTER (*p*-value)
*Fusion LLR*	2.897%	4.767%		*Fusion LLR*	3.556%	6.986%
Alpineon*	5.040%	7.076%		Alpineon*	7.982%	10.678%
L2F-EHU*	7.889%	8.191%		*Phonexia* +	8.364%	14.181%
*GIAPSI GSE*	8.332% (1.39 × 10^−8^)	8.382% (5.86 × 10^−9^)		*GIAPSI GSE*	10.643% (4.85 × 10^−4^)	13.107% (1.40 × 10^−10^)
*GIAPSI GSE* + *VTE*	8.496% (2.09 × 10^−5^)	8.631% (1.70 × 10^−3^)		L2F-EHU*	11.005%	17.266%
*Phonexia* +	9.601%	10.779%		*GIAPSI GSE* + *VTE*	11.016% (2.02 × 10^−1^)	13.150% (5.68 × 10^−18^)
*IDIAP*	9.960%	10.032%		*Mines-Telecom* +	11.429%	11.633%
*Mines-Telecom* +	10.198%	9.109%		*IDIAP*	12.011%	14.269%
L2F*	10.599%	11.129%		L2F*	13.484%	22.140%
*EHU*	11.310%	10.058%		CPqD*	14.348%	15.987%
CPqD*	11.824%	10.214%		*ATVS* +	16.836%	17.858%
*CDTA*	12.738%	19.404%		*EHU*	17.937%	19.511%
*ATVS* +	14.881%	15.429%		*CDTA*	3.556%	6.986%
*RUN* +	24.643%	22.524%		*RUN* +	7.982%	10.678%

*First row corresponds to results achieved by the fusion of all systems. Red, systems performing fusion of different systems. Orange, systems incorporating external/additional training data. Green, results achieved by the proposal presented in this article*.

## Discussion

In order to test the importance of an accurate front-end to better characterize a speaker, a complete system has been build based on the GMM–UBM classifier. The results achieved in the two presented scenarios show that the use of a gender-dependent extended biometry parameterization provides a more accurate description of the speakers than the one based on classical gender-independent MFCCs, thus confirming the hypothesis of the present study.

Although it is well known that male and female voices differ significantly and even some speaker recognition systems used gender-dependent classifiers, the application of a gender-dependent parameterization has not been explored previously. From the test carried out in this experiment, it is clear that not only the classifier system but the front-end system must be gender dependent in order to provide enhanced recognition rates.

Concerning the use of extended biometric information which complements classical parameters to provide an accurate characterization of speakers, we can perform a separate analysis on what we have called alternative parameters, and on the set of parameters extracted from the GSE and VTE.

Under alternative parameters, we have grouped the parameters related to energy of the frame (E), delta energy (ΔE), pitch (F0), and third formant estimate (F3). Energy and ΔE coefficients are typically used in SR systems as they constitute a heritage from the speech recognition area; however, we have verified that in most cases their use, combined with classical MFCCs, do not usually provide an improvement in terms of recognition rates. On the contrary, F0 and specially F3 (usually combined with some of the other alternative parameters) are extremely useful in most of the presented scenarios in order to precisely characterize speakers, and therefore to reduce recognition errors. Thus, the use of F3 is confirmed as an important contribution, as this parameter seems to be very relevant for speaker recognition tasks.

Regarding the use of information extracted from the VTE and GSE, we can extract interesting conclusions. First of all, we assume that information conveyed by the glottal source is not only closely related to physiological (and therefore biometric) characteristics of speakers, but also somewhat free from the influence of the message cast. For this reason, it should be more effective in order to characterize speakers, especially in text-independent scenarios. Complementary, the VTE provides information dependent on the phonetic content of the message. Therefore, the amount of training information needed to model the speaker is going to be larger than in the case of the glottal component as sufficient phonetic coverage will be required from the speaker. This requirement is not always possible to fulfill with the databases used in this research, and in many practical cases in the real world. As a result, the improvements achieved when using parameters extracted from this component are more limited than in the case of the GSE.

In this work, we have chosen to use a parameterization of the VTE and GSE in the form of MFCCs, which obviously masks any semantic meaning of the used parameters. We have ruled out the use of another set of parameters, which in theory should provide better results for different reasons. One reason is that, for instance a time-based (Gómez Vilda et al., 2009) set of parameters requires high quality sound recordings in order to be properly estimated. However, high quality is not usually present in many real application scenarios. Another reason is the additional problem that may appear when using parameters which convey specific semantics. Specifically, the expected benefit may be neutralized by two key factors: estimation error and estimation at unsuitable instants. In this sense, an additional problem arises regarding the value that must be assigned to these semantic parameters at the specific instant when their estimation is not adequate. Should we assign the last value? Maybe zero? Or maybe the mean value over some specific previous interval? For this reason, it may be useful to conduct a study on the effect of non-periodic parameters on the whole speaker recognition system.

The set of features presented in this work to characterize speakers have proven to be robust to channel (ALBAYZIN database consists of microphone recordings, while MOBIO database consists of cell phone recordings) and even language variability (ALBAYZIN database consists of Spanish spoken recordings, while MOBIO database consist of English spoken recordings). However, there are other sources of variability that may affect the SR system, which deserve further analysis. The feature set presented in this work to improve recognition rates intend to somehow model physical characteristics of speakers. Usually two unrelated speakers will have different physical characteristics thus leading to different parameter’s value. The question that arises now is: what happens when there is a strong link between two speakers? i.e., what happens in the case of siblings, monozygotic, and dizygotic twins? The latter case is attracting great interest as demonstrated by the research activity in this area (San Segundo and Gómez Vilda, 2013). Therefore, it seems interesting to evaluate the robustness of our proposal in scenarios like this, as some other biometric authentication methods such as face seem to fail.

Another key aspect affecting any SR system is robustness against temporal variations. Not only do we mean changes due to emotional states (joy, elation, sadness, anger, etc.) or temporal health issues, but also the changes that occur in the voice as a direct result of aging or neurological deterioration. It is clear that some age and neurological diseases have a clear effect in voice production. For this reason, it seems appropriate to carry out an analysis of the effect of aging in the biometric features proposed in the present study.

Finally, it must be noted that tasks related to the speaker recognition front-end have been relegated to a secondary plane when compared to the research interest in classification and normalization methods. Most speaker recognition systems keep on using MFCCs extracted from the power spectral density of speech as a whole (Khoury et al., 2013), even though these coefficients are inherited from the speech recognition area. Although offering a good performance, they are not as accurate as expected when characterizing speakers. In this sense, we can cite the use of ΔΔMFCC. These parameters are typically used in SR systems to characterize speakers; however, in the set of experiments we have run, they have not contributed to significant improvements to recognition rates. This allows us to state that when characterizing a speaker incorporating more number of parameters does not always result in recognition improvements. For this reason, once the high level of maturity in terms of classification techniques has been reached, it seems necessary to focus on the extraction process in order to improve the performance of these systems, selecting features that allow for an unambiguous way to characterize speakers. From this point of view, this work offers a new starting point for further research in the speaker recognition field.

## Conflict of Interest Statement

The authors declare that the research was conducted in the absence of any commercial or financial relationships that could be construed as a potential conflict of interest. The Review Editor Adam Vogel declares that, despite having collaborated on the Research Topic, Speech signal analysis with applications in biomedicine and the life sciences, with author Pedro Gómez-Vilda, the review process was handled objectively and no conflict of interest exists.
